# Local Reports of Epidemic and Endemic Diseases

**Published:** 1855-06

**Authors:** 


### LOCAL REPORTS OF EPIDEMIC AND ENDEMIC DISEASES,

*During the Months of March, April, and May* 1855.

The subjoined Report is compiled from data furnished specially for this journal by medical men in fourteen towns in England and Wales, viz :

| Place. | County. | Lat. | Long. | Observer. |
|---|---|---|---|---|
| Hastings - | Sussex - - | 50.51 N. | 0.34 E. | W. A. Greenhill, M.D. |
| Bridgewater - | Somerset - | 51. 8 N. | 3. 0 E. | Alfred Haviland, Esq. |
| Canterbury - | Kent - - | 51.17 N. | 1. 4 E. | G. Rigden, Esq. |
| Putney - - | Surrey - - | 51.23 N. | 0.12 W. | R. H. Whiteman, Esq. |
| Chatham - | Kent - - | 51.24 N. | 0.14 E. | F. J. Brown, M.D. |
| Wanstead - | Essex - - | 51.32 N. | 0. 2 W. | Fred. Collins, M.D. |
| Swansea - | Glamorgansh. | 51.38 N. | 3.50 W. | W. H. Michael, Esq. |
| Saffron Walden | Essex - - | 52. 2 N. | 0.17 E. | T. Spurgin, Esq.; H. Stear, Esq. |
| Thetford - | Norfolk - - | 52.26 N. | 0.45 W. | H. W. Bailey, Esq. |
| Nottingham - | Nottinghamsh. | 52.30 N. | 1.10 W. | T. Robertson, M.D. |
| Hawarden - | Flintshire - | 53.11 N. | 3. 2 W. | T. Moffat, M.D. |
| Alford - - | Lincolnshire - | 53.15 N. | 0. 6 E. | R. U. West, Esq. |
| Gainsborough | Lincolnshire - | 53.23 N. | 0.47 W. | D. Mackinder, M.D. |
| Liverpool - | Lancashire - | 53.24 N. | 2.59 W. | Thos. Bickerton, Esq. |

### QUARTERLY STATEMENT—No. II.

| Disease. | Where Observed. | At what Periods. |
|---|---|---|
| 1. Scarlatina - | Hastings - | Every week in quarter |
| „ | Canterbury - | Weeks end. Mar. 15; April 5, 12; May 3 |
| „ | Putney - - | Weeks ending May 17, 24, 31 |
| „ | Chatham - | Weeks end. Mar. 8, 15, 22; April 12; May 17, 31 |
| „ | Wanstead - | Weeks end. Mar. 8, 29; Apr. 12; May 24 |
| „ | Swansea - - | Every week in the period |
| „ | Saffron Walden | From March 18 to end of May |
| „ | Thetford - - | From March 18 to end of May |
| „ | Nottingham - | Weeks ending March 29; April 19 |
| „ | Hawarden - | Weeks end. Mar. 15, 29; Apr. 6; May 3 |
| „ | Alford - - | Weeks ending May 17, 24 |
| „ | Gainsborough - | During the whole of March |
| „ | Liverpool - | Every week throughout the quarter |
| 2. Measles - - | Hastings - - | Weeks end. Mar. 29; May 17, 24, 31 |
| „ | Bridgewater - | Week ending March 15 |
| „ | Putney - - | During Mar. and Apr., and weeks end. May 3, 17 |
| „ | Chatham - - | Weeks end. Apr. 12, & Apr. 26 to May 31 |
| „ | Swansea - - | Every week in period |
| „ | Thetford - - | Week ending May 19 |
| „ | Nottingham - | Weeks ending April 5, 26; May 3 |
| „ | Gainsborough - | Weeks end. April 12, 19, 26; May 3, 17 |
| „ | Liverpool - | Every week throughout quarter |

| Disease. | Where Observed. | At what Periods. |
|---|---|---|
| 3. Small-pox - | Hastings - - | Every week throughout period |
| " | Canterbury - | Weeks end. Mar. 8, & Mar. 29 to May 31 |
| " | Putney - - | March and three weeks in April |
| " | Chatham - - | During whole of March, and weeks ending May 10, 17, 24 |
| " | Saffron Walden | Weeks ending March 8 to April 12 |
| " | Nottingham - | Weeks ending March 29; April 19 |
| " | Liverpool - | Nearly all the quarter |
| 4. Hooping-cough | Hastings - - | Weeks end. Mar. 15; April 19; May 3, 17, 24, 31 |
| " | Bridgewater - | Weeks ending March 8, 15, 22 |
| " | Chatham - - | Weeks ending March 8 to April 5, and April 19 to May 3 |
| " | Swansea - - | Week ending April 3 |
| " | Nottingham - | Weeks ending March 8, 29; May 10 |
| " | Liverpool - | Nearly whole of quarter |
| 5. Croup - - | Hastings - - | Weeks ending March 29; May 3 |
| " | Putney - - | Week ending April 26 |
| " | Chatham - - | Weeks ending April 19; May 17 |
| " | Swansea - - | Weeks ending March 27, and April 17 to May 15 |
| " | Thetford - - | Week ending April 12 |
| " | Nottingham - | Weeks ending Mar. 8, 15, 22; April 5 |
| " | Liverpool - | Weeks ending April 5, 26; May 10 |
| 6. Catarrh - - | Bridgewater - | Weeks ending March 8, 15 |
| " | Putney - - | Weeks ending April 12, 19, 26 |
| " | Chatham - - | During whole of quarter |
| " | Wanstead - | Weeks ending March 8, 15, 22; April 12; May 3 |
| " | Swansea - - | Weeks end. Mar. 8, 15; May 17, 24, 31 |
| " | Saffron Walden | Every week |
| " | Thetford - - | Weeks ending March 8, 15; April 5, 12, 19; May 17, 24 |
| " | Nottingham - | Through quar. except week end. May 3 |
| " | Hawarden - | Weeks ending April 12; May 31 |
| " | Gainsborough - | Every week |
| " | Liverpool - | During most of the quarter |
| 7. Influenza - | Hastings - - | Week ending May 3 |
| " | Bridgewater - | Weeks end. Mar. 8, 15; Apr. 5; May 10, 17 |
| " | Putney - - | Weeks end. Mar. 8 to Apr. 5, & Apr. 19 |
| " | Chatham - - | Whole of March and April, and week ending May 17 |
| " | Wanstead - | Whole of March, and weeks ending April 5 and 19 |
| " | Swansea - - | Week ending May 31 |
| " | Saffron Walden | Every week in quarter |
| " | Thetford - - | Weeks ending March 8, 15, 22, and from April 12 to May 17 |
| " | Nottingham - | March and April, and weeks ending May 17, 24, 31 |
| " | Alford - - | Weeks ending from May 10 to 31 |
| " | Gainsborough - | Weeks ending Mar. 8, 29; April 5, 12; May 24, 31 |
| " | Liverpool - | During Mar. and week end. May 24 |
| 8. Erysipelas - | Hastings - - | Weeks ending March 8; May 3 |
| " | Bridgewater - | Weeks ending April 5, 12; May 3 |
| " | Canterbury - | During Apr. & weeks end. May 10, 17, 24 |

| Disease. | Where Observed. | At what Periods. |
|---|---|---|
| 8. Erysipelas - | Putney - - | Weeks ending May 10, 17 |
| „ | Chatham - - | Weeks ending March 8, 22, 29; April 12, 26; May 3, 17 |
| „ | Wanstead - | Weeks ending March 15, 22 |
| „ | Swansea - - | Weeks ending March 17, 31 |
| „ | Saffron Walden | Weeks ending Mar. 8, 15; May 10 |
| „ | Thetford - - | Weeks end. Mar. 15; Apr. 19; May 24, 31 |
| „ | Nottingham - | Weeks from Mar. 15 to April 12; May 3, 24, 31 |
| „ | Hawarden - | Week ending May 3 |
| „ | Alford - - | Week ending May 10 |
| „ | Gainsborough - | Weeks ending Mar. 8; April 5, 12, 19 |
| 9. Cholera - - | Chatham - - | Week ending May 31 |
| 10. Ague - - | Hastings - - | Weeks ending April 19; May 24, 31 |
| „ | Canterbury - | Weeks ending Mar. 8, 15, 22, and from April 5 to May 17 |
| „ | Chatham - - | Nearly every week in quarter |
| „ | Wanstead - | Weeks ending Mar. 29; Apr. 5, 12 |
| „ | Saffron Walden | Weeks end. Mar. 8; Apr. 19, 26; May 17 |
| „ | Nottingham - | Week ending May 17 |
| „ | Gainsborough - | Weeks ending April 26; May 3 |
| 11. Remittent Fever | Canterbury - | Weeks ending March 8, 15, 22; April 19; May 3 |
| „ | Putney - - | Weeks ending from Apr. 26 to May 31 |
| „ | Wanstead - | Weeks end. Mar. 29; Apr. 26; May 24 |
| „ | Swansea - - | Weeks ending April 19, 26 |
| „ | Thetford - - | Weeks end. Apr. 26; May 10, 24, 31 |
| „ | Nottingham - | Weeks ending Mar. 15; April 19 |
| „ | Hawarden - | Week ending March 15 |
| „ | Liverpool - | During March and April |
| 12. Diarrhœa - | Hastings - - | Weeks end. Mar. 18, 22; May 24, 31 |
| „ | Bridgewater - | Weeks ending April 5, 19 |
| „ | Putney - - | Weeks end. Apr. 19, 26; May 17, 24, 31 |
| „ | Chatham - - | Weeks ending Mar. 8, 15, 29; April 26; May 10, 17, 24 |
| „ | Wanstead - | Weeks ending Mar. 15, 29; May 3 |
| „ | Swansea - - | Weeks from March 22 to April 26; May 17, 24, 31 |
| „ | Saffron Walden | Week end. Mar. 15, & each week in May |
| „ | Thetford - - | Weeks ending March 8, 15, 22; April 5, 26; May 3, 10, 31 |
| „ | Nottingham - | Weeks ending March 8 to April 5; April 19; May 3, 17, 24 |
| „ | Hawarden - | During whole of quarter |
| „ | Alford - - | Weeks ending March 8, 15 |
| „ | Liverpool - | Nearly every week in quarter |
| 13. Dysentery - | Putney - - | Week ending April 26 |
| „ | Wanstead - | Week ending March 29 |
| „ | Swansea - - | Weeks ending April 12, 19 |
| „ | Saffron Walden | Week ending March 8 |
| „ | Nottingham - | Weeks end. from Mar. 29 to May 3, 31 |
| „ | Alford - - | Weeks ending March 8, 15; May 10 |
| „ | Gainsborough - | Week ending March 8 |
| „ | Liverpool - | Weeks end. Mar. 15; Apr. 5, 12; May 3 |
| 14. Typhus - - | Hastings - - | During March, and weeks end. April 19, 26; May 3, 10, 31 |
| „ | Bridgewater - | Weeks ending Mar. 15, 22; April 12 |

| Disease. | Where Observed. | At what Periods. |
|---|---|---|
| 14. Typhus - - | Canterbury - | Weeks end. from Apr. 12 to May 24 |
| „ | Putney - - | Weeks ending Mar. 15, 29; Apr.; 19 May 24 |
| „ | Chatham - - | Weeks ending Mar. 29; April 19, 26; May 10, 17, 24 |
| „ | Thetford - - | Weeks ending Mar. 8; Apr. 5, 19; May 10, 24 |
| „ | Nottingham - | Weeks ending Mar. 15, 29; Apr. 26; May 10, 17, 24 |
| „ | Gainsborough - | Weeks from April 19 to May 31 |
| „ | Liverpool - | Weeks end. Mar. 15; Apr. 26; May 3 |
| 15. Puerperal Fever | Bridgewater - | Week ending March 8 |
| „ | Nottingham - | Week ending May 10 |
| „ | Liverpool - | Week ending May 31 |
| 16. Carbuncle - | Bridgewater - | Week ending April 5 |
| „ | Canterbury - | Nearly every week in quarter |
| „ | Chatham - | Weeks ending March 22; May 17 |
| „ | Wanstead - | Weeks ending April 26; May 24 |
| „ | Swansea - - | Weeks ending March 29; April 19 |
| „ | Saffron Walden | Weeks ending March 29; May 3, 24 |
| „ | Thetford - - | Weeks ending April 26; May 31 |
| „ | Nottingham - | Weeks end. Mar. 8; Apr. 12; May 3, 31 |
| „ | Alford - - | Week ending March 8 |
| „ | Gainsborough - | Weeks ending Mar. 22, 29; April 5 |
| „ | Liverpool - | Weeks end. Mar. 15, 22; Apr. 12, 19 |

### ADDITIONAL OBSERVATIONS.

*Hastings.*—Dr Greenhill makes the following remarks: "Scarlatina was prevalent throughout the whole of the spring, and in all parts of the borough; generally of a mild type, the malignant cases being chiefly caused by want and neglect. Thirteen deaths occurred, which is very far above the average number during the spring of the previous ten years.

"Small-pox was prevalent throughout the spring, during March in the western parishes, during April and May chiefly in the eastern. Many cases occurred after vaccination, in which the disease was mild in type: one patient had small-pox last autumn, *i. e.* two attacks within nine or ten months. Four deaths occurred, which is above the average.

"Two deaths occurred from hooping-cough, which is less than the average.

"Several cases of typhus occurred (using the word in the extended signification given to it in the Registrar-General's statistical nosology), chiefly in the western parishes. There were three deaths, which is about the average number.

The statement is compiled from returns furnished by the East Sussex Medico-Chirurgical Society.

*Bridgewater.*—Mr. Haviland furnishes some very complete medico-meteorological observations.

"*March* 8.—Amount of ozone during the week, 20. Wind

N.W. and E.S.E. Range of barometer, 1 in. 15. Ditto thermometer, 17°; highest at 9 A.M. 51; lowest at 9 A.M. 34. Windy at the beginning of the week, hence the amount of ozone.

"Deaths from bronchitis, 3; old age, 2; puerperal fever, 1; dice, 1; unknown, 1; total, 8.

"Mar. 15.—Ozone, 23. Wind S.E. N.W. The hurricanes from the latter quarter. Range of barometer, 1.60 in. Of thermometer, 9°; lowest at 9 A.M. 34°. 43°.

"Deaths from bronchitis, 1; fever, 1; convulsions, 1; total, 3.

"— 22.—Ozone, 13. Wind N.W. and E.N.E. Range of barometer, 1.4 in. On the 15th the barometer was lower than it had been during the half-year; it stood at 28.16 in. Range of thermometer, 13°; highest, 48°; lowest, 35°.

"Deaths from bronchitis, 2; influenza, 1; phthisis, 1; diseased bladder, 1; old age, 2; fever, 1; paralysis, 1; epilepsy, 1; mesenteric disease, 1; total, 12.

"— 29.—Ozone, 6. Calm generally. Wind N.E. and N.W. Range of barometer, 0,80 in. Range of thermometer, 7°; highest, 42°; lowest, 35°.

"Deaths: bronchitis, 2; old age, 1; gout, 1; phthisis, 2; tabes, 1; mesenteric disease, 2; neuralgia, 1; unknown, 2.

"*April* 5.—Ozone, 7. Wind N.W. and N.E. Range of barometer, ? Range of thermometer, ? Frost and snow.

"Deaths from phthisis, 3; diarrhœa, 1; dentition, 2; phrenitis, 1; total, 7.

"— 12.—Ozone, 52. Strong wind all the week from N.W. Range of barometer, 0.80 in. Range of thermometer, 8°; highest, 55°; lowest, 47°. Great mortality among cattle and horses where exposed to N.W. aspect.

"Deaths from phthisis, 4; fever, 1; old age, 1; debility, 1; convulsions, 1; disease of the heart, 1; total, 9.

"— 19.—Ozone, 13. Wind N.W. and N.E. Range of barometer, 1.00 in. Range of thermometer, 7°; highest, 55°; lowest, 48.

"Deaths from phthisis, 3; disease of the stomach, 1; unknown, 1; total, 5.

"— 26.—Ozone, 2. Wind N.E. Range of barometer, 0.20 in. Of thermometer, ? Fine throughout the week. Neuralgia prevalent.

"Death from hydrocephalus, 1; total, 1.

"*May* 3.—Ozone, 10. Wind N.E. Range of barometer, 0.60 in. Of thermometer, 11°; highest, 54°; lowest, 43°.

"Deaths from bronchitis, 1; disease of the lungs, 1; phthisis, 1; old age, 2; pneumonia, 2; lumbar abscess, 1; total, 9.

"— 10.—Ozone, 27. Wind N.E. and N.W. Range of barometer, 0.28 in. Of thermometer, 70°; highest, 55°; lowest, 47.

"Deaths from asthma, 1; rheumatic fever, 1; paralysis, 1; syncope, 1; hydrocephalus, 1; total, 5.

"— 17. Ozone, 21. N.W. Range of barometer, 0.60 in. Of thermometer, 10°; highest, 54°; lowest, 44°. Deaths from bronchitis, 1; phthisis, 1; old age, 1; convulsions, 2; total, 5.

"May 24.—Ozone,30. Wind S.E. Range of barometer, 0.47 in. Of thermometer, 8°; highest, 57°; lowest, 49°.

"Deaths from phthisis, 1 ; disease of the brain, 1 ; total, 2.

" — 31.*—Ozone, 23. Wind E.S.E. and N.E. Range of barometer, 0.35 in. Of thermometer, 18°; highest, 63°; lowest, 45°.

"Deaths from phthisis, 3; convulsions, 1; total, 4."

*Canterbury.*—Dr. Rigden writes that, "Small-pox has been the prevailing epidemic during the last three months ; several cases have occurred after vaccination, they have been generally of a modified form. I have not had nor heard of a case of secondary small-pox; forty-five cases have been under my treatment, of which number four have proved fatal—all of a confluent and unmodified character."

*Putney.*—Mr. Whiteman writes: "Small-pox prevailed to a somewhat serious extent during March and April. Two adult persons died of the disease who had never been vaccinated. Indeed, out of a dozen cases, two only were found with well marked cicatrices. All the cases occurred in adults ; and, with the exception of the two who had been vaccinated in infancy, the patients who came under my care had the disease in its most severe form. Two or three cases of scarlet fever have also come under treatment; one of a very malignant type. Measles have been very prevalent but of a mild character. Remittent fever occurred during May, chiefly amongst children.

"Vegetation has been very backward, and in some situations disease has threatened the potato crop."

*Chatham.*—Dr. Brown says: "In the first two months of this quarter, influenza and hooping cough affected a great number of individuals. In the latter half of the quarter, measles have prevailed most extensively. There have been many instances of second attacks, as well in children as in adults.

"A few cases of mumps have occurred in schools. Cases of paronychial inflammation have been abundant.

"Intermittent fever has attacked several parturient women, but no deaths have occurred.

"The continued fever that has been witnessed has been the typhoid and the febricula, exclusively of typhus and relapsing fever. The cases of continued fever have not been numerous.

"There have been several instances of spring diarrhœa, accompanied by vomiting. In one case the ejesta were serous, and the patient's condition analogous to that of cholera. The subject of the attack, a child residing in an alley in Rochester, was sickened by the smell that was occasioned by a drain that was in process of clearance.

"A case of well-marked scorbutus occurred in a poor woman, aged 54 years, residing in Chatham.

---

* All the barometric and thermometric observations were taken about nine a.m.

"The weather has been very cold; and the wind has blown 42 days out of 92 from the east and north. Vegetation has been backward.

*Wanstead.*—Dr Collins says: "Neuralgia and rheumatism have prevailed during the quarter, and diseases have put on an intermittent type. I have had eight cases of ague (during the quarter) which is singular, as ague is rare here. I do not recollect having met with a case in this neighbourhood before."

*Swansea.*—Mr. Michael states: "In April, many sheep died: the weather was extremely cold, and the herbage very scanty. In May, vegetation was at least a month behind the average: many wild flowers were not in bloom until the middle of the month. There was great prevalence of measles; almost every house in the town was affected. In April, the cases were very severe; the breathing was extremely rapid; and the eruption, mulberry coloured, followed by gangrene of the lips, cancrum oris, exfoliation of the jaw, &c., &c. The cases generally recovered, but were followed by very great depression. As the weather became warmer, a much milder form of the disease prevailed. The scarlatina was mild; in a few cases there was scarlatina anginosa with ozæna."

*Saffron Walden.*—Mr. Spurgin writes as follows: "The season is unusually backward, and vegetation only now beginning to make any progress. Corn in general, and wheat in particular, looks healthy. The dryness of the atmosphere has been favourable to animal health, where much exposed to its influence. Pigs have suffered from measles.

"Scarlatina has been very severe, and in several cases fatal; three, terminating in death, have occurred in my own practice. Small-pox has also been severe, and four cases have ended fatally, all of which are said to have been unprotected by vaccination. The wind has been generally north-east, and occasionally dry and cold.

"The small-pox was brought, I understand, from London by an individual to his family, and, wishing to conceal it, the disease was allowed to spread to surrounding families, and from them to others before any active measures were taken for separation. Altogether, I hear there were about forty-eight cases, four of which were fatal, but none of these had been protected by vaccination. Several had been vaccinated, who had it mildly.

"I had no cases myself, in the town, and only one at Littlebury, the origin of which was obscure, although I think it was carried from Walden to the patient. This case did not extend itself; the house was completely isolated, and all communication cut off. The patient had been vaccinated; was nursed entirely by his sister, and attended by a servant, both of whom had been vaccinated, but both escaped infection, although the disease was severe. Two other individuals in the house had been protected by small-pox in early life".

Mr. Stear states: "Scarlet fever and small-pox have been very prevalent during the quarter. Little rain had fallen; and the wind has been generally north-east. Fever of a low typhoid kind has affected cattle to a considerable extent. Vegetation has been very backward: the crops, generally speaking, are healthy."

*Thetford.*—Mr. Bailey writes: "The temperature of the last three months has, throughout, been from 4° to 7° below the average of the corresponding months of the last three years. Vegetation very backward, and the foliage of some trees not fully out. This continued state of cold, together with the northerly and easterly winds, have produced more sickness than I have observed for many years in this locality. Scarlet fever has been epidemic, attacking whole families in all parts of our district. The disease has been of a mild character, but in many cases followed by dropsical effusions. No fatal case has occurred. In the immediate neighbourhood measles have been prevalent, although only one case has come under my notice. The eruption was nearly confluent, and symptoms very severe. In the same parish several families were attacked with the disease. One case of croup occurred in a child two years of age, from being exposed, late at night, to a cold easterly wind: although active means were resorted to, death took place suddenly in two days. Catarrh and influenza are still very prevalent, attacking all grades of society. Erysipelas of the face and head affected three persons very severely, one of whom, being in perfect health, visited her friend who was affected with the disease, and in a few days had an attack of it in the face and head, of a most severe kind, attended with delirium and fever. Did this arise from infection? It is the only case I ever observed which might be attributed to that cause. In hospitals it is generally supposed to be infectious. In our union-house and among the labouring classes, ague and remittent fever have come under our observation, arising from imprudent exposure and wet. These have quickly given way to quinine. Diarrhœa has been more prevalent within the last two months, chiefly among children and some adults, and appears quite epidemic. I have never known so many cases of fever assuming the typhoid type. These do not occur in any peculiar locality. In this town we are particularly dry, the houses are clean, and the authorities attend strictly to the hygienic state of the town. They, therefore, appear to arise from an epidemic state of the atmosphere. Labourers are those chiefly attacked, and especially those who are unable to supply themselves with the common necessaries of life. These cases run on to the third and fourth week, leaving the system much shaken and prostrated; in one a carbuncle has formed in the neck, and in others boils in various parts. Bronchitis in children has been very general the last three weeks, requiring constant watching, and in some cases active treatment.

"The report which I have had from our veterinary surgeon is, that pleuropneumonia has been prevalent among the cattle in this neighbourhood, and violent inflammation has attacked horses both in the chest and bowels—fatal in many instances. Also strangles have attacked horses. Coughs have been general among them during the last two months. Considerable mortality has taken place in sheep from the severity of the weather during the time of lambing. Within the last few days vegetation has made rapid progress. Summer seems to come upon us all at once, and there appears a prospect of a good fruit year.

"The following are the total of cases from the registrar's book in his district.

| *March.* | | *April.* | | *May.* | |
|---|---|---|---|---|---|
| Debility | 4 | Debility | 2 | Hepatitis and jaundice | 1 |
| Decay of nature | 2 | Diseased liver | 2 | Infusion on the brain | 1 |
| Diabetes | 1 | Gangrena senilis | 1 | Decay of nature | 3 |
| Pneumonia | 4 | Fever | 1 | Debility | 6 |
| Nephritis | 1 | Ascites | 1 | Pneumonia | 1 |
| Burn | 1 | Cephalitis | 1 | Emphysema of lungs | 1 |
| Scrofulous abscess | 1 | Decay of nature | 3 | Phthisis pulmonum | 3 |
| Bronchitis | 2 | Convulsions | 1 | Bronchitis | 1 |
| Phthisis pulmonum | 1 | Cephalœa | 1 | Inflammation of brain | 2 |
| Railway accidents | 2 | Pneumonia | 1 | Paralysis | 1 |
| Hydrocephalus | 1 | Apoplexia | 1 | Atrophy | 1 |
| Diseased abd. viscera | 2 | Jaundice | 1 | Influenza | 3 |
| | | Influenza | 1 | Secondary syphilis | 1 |
| | | Uteritis | 1 | Inflammation of chest | 2 |
| | | Phthisis pulmonum | 1 | | |
| | | Diseased prostate | 1 | | |
| | 22 | | 20 | | 27 |

"Total, 69."

*Nottingham.*—Dr. Robertson writes: "The quarter just finished has been principally remarkable for its great severity. In March (according to Mr. E. A. Lowe's register), the temperature was only above 50° on 7 days in the month; and there was frost on 29 nights. Snow fell on the 11th, hail on the 18th, sleet on the 22nd, hail and snow on the 23rd. The wind was mostly N. E., and the amount of rain 1.13 inches. During the whole month, except between the 24th and 29th, ozone was constantly indicated. April was also a very cold month; frost occurring on 19 nights. The amount of rain was .973 of an inch; wind variable. There was hail on the 10th, and much ozone during the month. May was inclement until the 18th, when warmer weather commenced. There was 16 frosty nights; $1\frac{3}{4}$ inches of rain. Wind changeable. Hail on the 1st, 8th, and 30th; snow on the 3rd and 4th. There was much ozone all the month. The swallows arrived on the 10th of April, and the cuckoo on the 10th of May. On the 31st of May an extraordinary number of swallows were found dead in the neighbourhood; a great number of sheep have also been lost during the month in this and the adjoining counties.

"Mr. Taylor informs me there has been no particular epidemic among the cattle; stomach and bowel complaints being the most prevalent. Owing to the late spring and scarcity of hay, many animals have had to be fed on straw; and constipation, requiring strong purgatives, has been a general result. In one case of Mr. Taylor's, rupture of the stomach occurred.

"The epidemics noted in the list do not require any particular remark. As will be seen, the respiratory organs have been principally affected, except in April, when diarrhœa and dysentery also prevailed. The eruptive fevers have all been seen, but not to any great extent. Mr. M. H. Higginbottom, under whose care the case of puerperal fever occurred, writes to me that the patient lived in a low and unhealthy locality. She was attended by a midwife, and Mr. H. did not see her for several days after her confinement. The atmosphere of the room, he says, was close and fetid in the extreme. He is not aware of any cases of erysipelas having occurred in the neighbourhood, or whether the midwife had attended other cases in which fever had supervened. Great improvement has lately been effected in the sanitary state of this town, to which I hope to be able to allude at a future time."

*Alford.*—Mr. West says: "During the past quarter we have had continual easterly winds, and very cold, dry weather. Influenza has been prevalent, and a good deal of an adynamic kind of pneumonia, with great intolerance of bleeding. I am of opinion that pneumonia occasionally prevails epidemically to such an extent as would justify a column being given to it in the Local Report. I have been called in to three of these cases, all attended with pain, rusty sputa and crepitation, with hurried respiration."

*Gainsborough.*—Dr. Mackinder says: "The past quarter has been healthy, considering the very severe weather which we have had. Catarrh has existed throughout; influenza during March and the end of May, and is now all but epidemic. I have had but three cases of measles, which were imported, and all in one house. In the first I was summoned on the 7th April, the second on the 28th April, and the third on the 14th May. There were but two cases of scarlatina, and but a few cases of mild fever. Mr. Sharp, the resident-surgeon of our Dispensary, informs me that 'there have been eleven cases of fever, two of which were the cases of scarlatina mentioned above, and sixteen cases of bronchial affections, many of which put on a severe form, ending in typhoid fever.' Of the other nine cases of fever, five were mild. Rheumatism and tic have been more than usually prevalent. I had but one case of carbuncle, one of erysipelas, and one of ague, which was a very mild one, though I have the charge of the workhouse to a union district of 17,437 acres, with a population of 9,616."

Dr. Mackinder also forwards a meteorological report, drawn up by Mr. Dyson, a member of the Meteorological Society.

"The weather, with the exception of the last week of the quarter ending May 26th, like that of the whole year from the 10th of January, was unusually severe; the temperature of every week, and with rare exceptions of every day, being below the average of thirty-eight years, over which registered and authentic observations have been made. Snow fell on nearly every day from the 15th of January to the last week in February. The severity of the storm continued six weeks: on the 15th of February the thermometer fell to 5°, and on the 17th to 7° , or 20° and 25° respectively below the freezing point of Fahrenheit's scale. The wind blew from the N. and E., or some combination of these points, on 103 days out of 135. Shrubs, which usually stand untouched during the winter in this latitude, such as the laurel and laurestinus, were almost entirely destroyed. The Trent was frozen over for a month. Water from melted snow amounted to ·49 inches in January; 1·92 inches in February; and rain in March 1·31; in April ·41; and in May, to the 26th, 1·05 inches; total, 5·28 inches, less than one-fourth of the average annual fall. The sky was entirely covered with cloud on more than half the number of days in the quarter. The hottest day of the year was on Saturday the 26th. The thermometer reached 80° in the shade. Ozone was largely developed at intervals during the quarter, particularly on the 2nd of March, 7th, 16th, and from the 19th to 24th. From the 7th to 10th of April, and from the 24th to the end of the month; on May 3rd, 11th, 13th, 14th, 19th, 21st, and 26th. The first swallow was seen on April 13th, and the cuckoo first heard on the 23rd, both being solitary instances. Vegetation was unusually late. The early part of the month of May was bitterly cold, and hail storms were prevalent. The lilac was not in flower, nor the oak and ash in leaf till the last week of the month. Cereal crops look well; but the meadows are scanty, and promise a thin hay-crop."

*Liverpool.*—Mr. Bickerton writes that scarlatina prevailed extensively in March. On May 24th, measles were rather common: scarlet fever was declining. During April and May bronchitis prevailed considerably. An erratic case of erysipelas occurred in a suckling infant. Common continued fever was frequent during the quarter. Acute rheumatic fever prevailed to a considerable extent. Pulmonary consumption was more than usually fatal in March and April.

